# Benefit of hydrocortisone, thiamine, and vitamin C for patients with sepsis or septic shock? Too early to draw conclusions

**DOI:** 10.1186/s13054-020-03153-5

**Published:** 2020-07-14

**Authors:** Rui Shi, Hongtao Tie

**Affiliations:** 1 Service de Médecine Intensive – Réanimation, Hôpital Bicêtre, AP-HP, Université Paris-Saclay, Le Kremlin-Bicêtre, France; 2 INSERM UMR_S999 LabEx - LERMIT, Hôpital Marie-Lannelongue, Le Plessis Robinson, France; 3 Department of Cardiothoracic Surgery, The First Affiliated Hospital of Chongqing Medical University, Chongqing, 400016 China

**Keywords:** Hydrocortisone, Thiamine, And vitamin C, Sepsis or septic shock, Meta-analysis

Dear Editor,

Previous studies demonstrated that a combination of hydrocortisone, vitamin C, and thiamine (HVT) is a promising adjuvant treatment for sepsis and septic shock, with decreased mortality and improved resolution of disease [1]. However, recently published prospective, randomized controlled trials (RCTs) did not support this finding [2]. Therefore, we performed a meta-analysis to evaluate the efficacy of HVT treatment for patients with sepsis or septic shock.

This meta-analysis was performed strictly following the Preferred Reporting Items for Systematic Reviews and Meta-analysis (PRISMA) statement [3]. The primary outcomes were mortality, decrease of sequential organ failure assessment score from baseline (Delt SOFA), and time of vasopressors use. Relative risk (RR) with 95% confidence intervals (CIs) and weighted mean differences (WMDs) with 95% CIs were used. Meta-analyses were performed using a random-effects model by RevMan version 5.1.

Four RCTs with 528 patients and five cohorts involving 412 patients were included, as described in Table 1. As shown in Fig. 1, pooled results from RCTs showed that HVT has no benefit on mortality (RR 0.92, 95%CI 0.69 to 1.24, *p* = 0.59; *I*^2^ = 0%, *P*_*H*_ = 0.69), while it was associated with a significant decrease of SOFA (Delt SOFA: WMD − 1.02, 95%CI − 1.31 to − 0.73, *p* < 0.001; *I*^*2*^ = 0%, *P*_*H*_ = 0.80) and reduction in time of vasopressors use (WMD − 21.77 h, 95%CI − 29.26 to − 14.29, *p* < 0.001; *I*^2^ = 0%, *P*_*H*_ = 0.4). Overall results from cohorts revealed that HVT could significantly reduce mortality (RR0.46, 95%CI 0.25 to 0.86, *p* = 0.01; *I*^2^ = 75%, *P*_*H*_ = 0.001) and SOFA score (Delt SOFA: WMD − 2.21, 95%CI − 4.22 to − 0.20, *p* = 0.03; *I*^2^ = 81%, *P*_*H*_ = 0.005), but not the duration of vasopressors use (WMD 1.11 h, 95%CI − 59.60 to  61.82, *p* = 0.97; *I*^2^ = 98%, *P*_*H*_ < 0.001). No differences in intensive care unit (ICU) length of stay and hospital length of stay between the HVT and the control group were observed.

**Table 1:** Baseline characteristics of the included studies

Study ID	Country	Study design	No. (HVT/Con)	Patient	Intervention	
					HVT group	Control
Chang 2020, PMID: [32243943]	China	Single-blinded RCT	40/40	Adult patients with sepsis or septic shock and procalcitonin PCT ≥ 2 ng/mL	- IV hydrocortisone (50 mg every 6 h for 7 days) - IV vitamin C (1.5 g every 6 h for 4 days) - IV thiamine (200 mg every 12 h for 4 days) - Standard care	- Placebo (normal saline) - Standard care
Fujii 2020, PMID: [31950979]	Australia, New Zealand, Brazil	Multicenter, open-label RCT	107/104	Adult patients with septic shock	- IV hydrocortisone (50 mg every 6 h for a maximum of 7 days) - IV vitamin C (1.5 g every 6 h for a maximum of 10 days) - IV thiamine (200 mg every 12 h for a maximum of 10 days) - Standard care	- IV hydrocortisone (50 mg every 6 h) - Standard care
Igelesia 2020, PMID: [32194058]	USA	Double-blinded RCT	68/69	Adult patients with sepsis or septic shock	- IV hydrocortisone (50 mg every 6 h) - IV vitamin C (1.5 g every 6 h) -IV thiamine (200 mg every 12 h) for a maximum of 4 days - Standard care	- Placebo (normal saline) - Standard care
Wani 2020, PMID: [31990246]	India	Open-label RCT	50/50	Adult patients with sepsis or septic shock and serum lactate level of > 2 mmol/L	- IV hydrocortisone (50 mg q 6 hourly for 7 days or until ICU discharge) - IV vitamin C (1.5 g every 6 h for 4 days or until discharge) - IV thiamine (200 mg q 12 hourly for 4 days or until discharge) - Standard care	- Standard care alone
Litwak 2019, PMID [30970560]	USA	Retrospective cohort study	47/47	Adult patient with septic shock	- IV hydrocortisone (200–300 mg every day) - IV vitamin C (1.5 g every 6 h) - IV thiamine (200 mg every 12 h) for 4 days - Standard care	- Standard care and/or IV hydrocortisone
Marik 2017, PMID: [27940189]	USA	Retrospective cohort study	47/47	Adult patients with severe sepsis or septic shock and PCT > 2 ng/mL	- IV hydrocortisone (50 mg every 6 h for 7 days or until ICU discharge) - IV vitamin C (1.5 g every 6 h for 4 days or until ICU discharge) - IV thiamine (200 mg every 12 h for 4 days or until ICU discharge)	- IV hydrocortisone (50 mg every 6 h) - Standard care
Mitchell 2019, PMID: [31469984]	USA	Retrospective cohort study	38/38	Adult patients with severe sepsis or septic shock	- IV hydrocortisone (50 mg every 6 h, 100 mg every 8 h, or 10 mg per h for 7 days) - IV vitamin C (1.5 g every 6 h for 4 days) - IV thiamine (200 mg IV every 1 for 4 days) - Standard care	- IV hydrocortisone - Standard care
Sadaka 2019, PMID: [31315499]	USA	Retrospective cohort study	31/31	Adult patients with septic shock	- IV hydrocortisone (50 mg every 6 h for 7 days) - IV vitamin C (1.5 g every 6 h for 4 days) - IV thiamine (200 mg every 12 h for 4 days) - Standard care	- Standard care alone
Wald 2020, PMID: [31916841]	USA	Retrospective cohort study	43/43(a)/43(b)	Pediatric patients with septic shock	- IV hydrocortisone (50 mg/m^2^/day divided every 6 h) - IV vitamin C (30 mg/kg/dose every 6 h for 4 days; maximum 1.5 g/dose) - IV thiamine (4 mg/kg/day for 4 days; maximum 200 mg/dose) - Standard care	a) Hydrocortisone only; b) standard care alone

*PMID* PubMed unique identifier; *RCT* randomized controlled trial; *HVT* hydrocortisone, vitamin C, and thiamine; *Con* control; *PCT* procalcitonin; *IV* intravenous; *h* hour

**Fig. 1:**
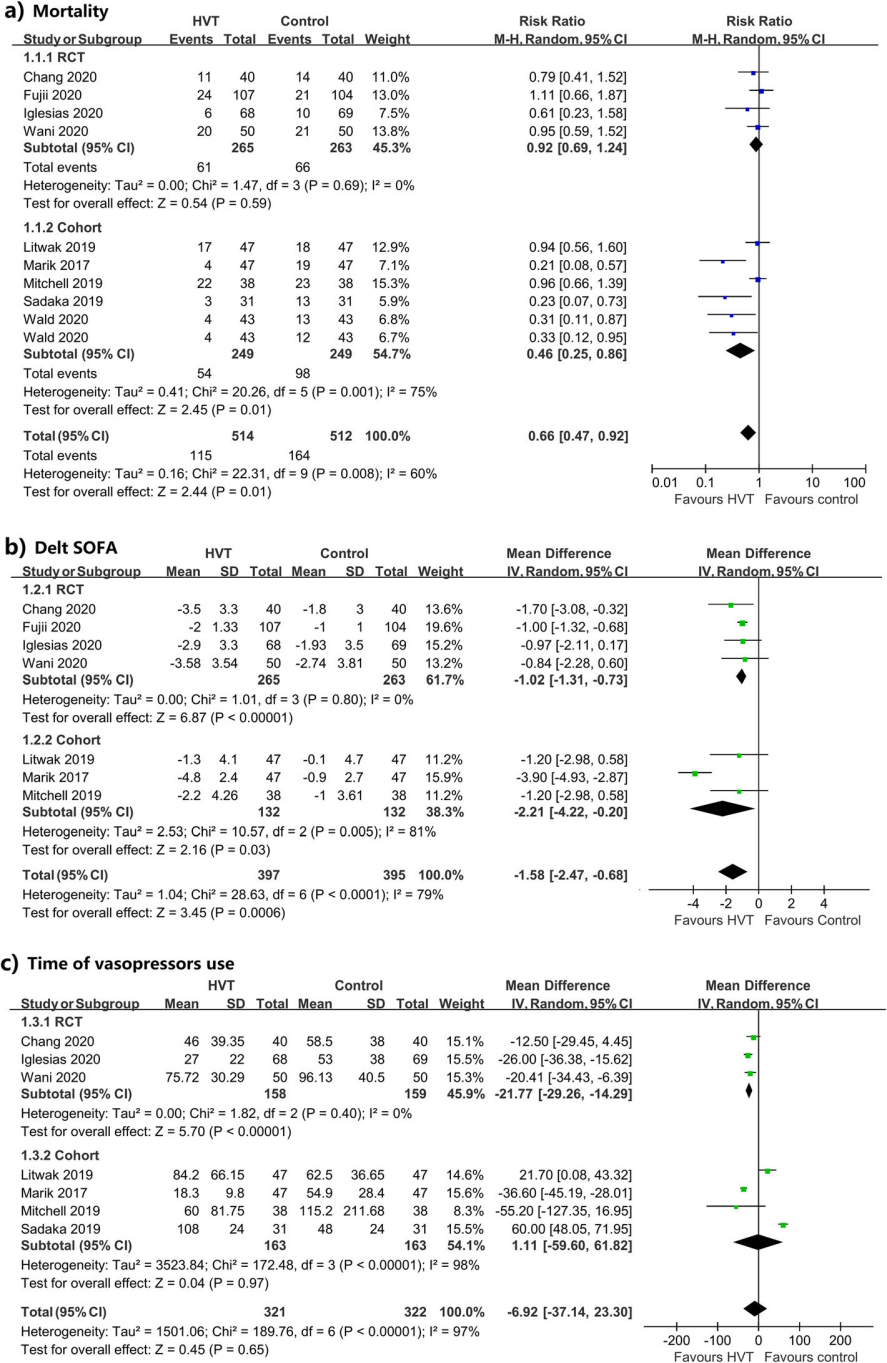
Forest plots for the primary outcome. **a** Mortality. **b** Delt SOFA. **c** Time of vasopressors use

Our study suggested that HVT has potential beneficial effects. A significant reduction in SOFA score was observed, although no benefit of mortality in the pooled effect of RCTs. Since it is a valuable end-point reflecting the disease process and also a surrogate marker for mortality, our meta-analysis of the four small RCTs might be inadequate to detect a mortality benefit. Additionally, the improved resolution of shock from RCTs also supports HVT use.

A generally recognized concept is that the HVT could synergistically restore the dysregulated immune system, oxidative mitochondrial function, and energy production [4]. However, current clinical practice regarding the HVT strategy is still debating. Besides the potential synergistic beneficial effects, the arguments supporting the use of HVT include low risk, low cost, and easy availability. Minor clinical side effects, such as hyperglycemia, hypertension, and hypernatremia induced by hydrocortisone [5], might occur but are insignificant and easily managed in ICU.

Some limitations merit consideration. Sample sizes in RCTs are small, and pooled effects on different outcomes are inconsistent. Though potential bias in cohort studies, the pooled result of cohort studies in our study supported and consolidated the findings from RCTs. Additionally, hydrocortisone was not systematically used for control groups in RCTs and cohorts. It is thus questionable to determine the benefit is from the synergistic effect of HVT or corticosteroid only, since the beneficial effect of corticosteroid sole has been well established [6]. Besides, other clinical heterogeneities, such as the timing of HVT and severity of the disease, should also be regarded. However, the data sparseness of included studies limited our subgroup analysis for further exploration.

In conclusion, the beneficial findings of our study support that HVT remains an attractive choice for sepsis and septic shock, while results from large-scale RCTs are still expected before a definite conclusion, especially in terms of the timing of HVT and the severity of sepsis.

## Data Availability

The datasets used and analyzed during the current study are available from the corresponding author on reasonable request.

## Abbreviations

HVT: Hydrocortisone, vitamin C, and thiamine
RCTs: Randomized controlled trials
WMDs: Weighted mean differences
RR: Relative risks
CIs: Confidence intervals
